# Interments in the City and Liberties of Philadelphia, from the 17th of July, to the 24th

**Published:** 1841-07-31

**Authors:** 


### HEALTH OF THE CITY

Interments *in the City and Liberties of Philadelphia, from the 17th of July, to the 24th.*

| Diseases. | Adults. | Children. |
|---|---|---|
| Asphyxia, | 0 | 1 |
| Apoplexy, | 3 | 0 |
| Affection of spine | 1 | 0 |
| Casualties, | 1 | 2 |
| Croup, | 0 | 2 |
| Congestion of Lungs, | 1 | 0 |
| Cholera Morbus, | 2 | 0 |
| Consumption of the lungs, | 11 | 3 |
| Convulsions, | 0 | 6 |
| Diarrhœa, | 1 | 6 |
| Dropsy, Abdominal, | 0 | 1 |
| —— Head, | 0 | 6 |
| Disease of Brain, | 0 | 2 |
| Drowned, | 4 | 1 |
| Dysentery, | 1 | 2 |
| Debility, | 0 | 9 |
| Fever, | 1 | 0 |
| —— Typhus, | 3 | 0 |
| —— Hectic, | 0 | 1 |
| Hæmorrhage from Uterus, | 1 | 0 |
| Inflammation of the Brain, | 0 | 5 |
| —— Bronchi, | 1 | 1 |
| —— Lungs, | 1 | 0 |
| —— Stomach, | 1 | 0 |
| —— Stomach and Bowels, | 1 | 1 |
| —— Bowels, | 0 | 3 |
| —— Breast, | 0 | 1 |
| —— Umbilical Cord, | 0 | 1 |
| Intemperance, | 2 | 0 |
| Inanition, | 0 | 3 |
| *Carried forward,* | 36 | 57 |

| Diseases. | Adults. | Children. |
|---|---|---|
| *Brought forward,* | 36 | 57 |
| Marasmus, | 0 | 7 |
| Measles, | 0 | 3 |
| Mania a Potu, | 1 | 0 |
| Neglect, | 0 | 1 |
| Old age, | 2 | 0 |
| Palsy, | 1 | 0 |
| Phlebitis, | 1 | 0 |
| Retention of Urine, | 0 | 1 |
| Scrofula, | 0 | 1 |
| Small pox, | 1 | 5 |
| Still-born, | 0 | 5 |
| Summer Complaint, | 0 | 45 |
| Tabes Mesenterica, | 0 | 1 |
| Ulcerated Sore Throat, | 0 | 1 |
| Total, 169— | 42 | 127 |

Of the above, there were under 1 year, 75

| | |
|---|---|
| From 1 to 2 | 32 |
| 2 to 5 | 14 |
| 5 to 10 | 4 |
| 10 to 15 | 1 |
| 15 to 20 | 1 |
| 20 to 30 | 11 |
| 30 to 40 | 10 |
| 40 to 50 | 8 |
| 50 to 60 | 1 |
| 60 to 70 | 4 |
| 70 to 80 | 3 |
| 80 to 90 | 4 |
| 90 to 100 | 1 |
| Total, | 169 |

Of the above there were 6 from the almshouse, 20 people of colour, and 3 from the country, which are included in the total amount.

